# Aphrodisiac Activity of* Eulophia macrobulbon* Extract on Erectile Dysfunction in Male Aged Rats

**DOI:** 10.1155/2018/6217029

**Published:** 2018-07-09

**Authors:** Watcharaporn Preedapirom, Kanokwan Changwichit, Piyarat Srisawang, Kornkanok Ingkaninan, Pornnarin Taepavarapruk

**Affiliations:** ^1^Department of Physiology, Faculty of Medical Science, Naresuan University, Phitsanulok 65000, Thailand; ^2^Department of Pharmaceutical Chemistry and Pharmacognosy, Faculty of Pharmaceutical Sciences and Center of Excellence for Innovation in Chemistry, Naresuan University, Phitsanulok 65000, Thailand

## Abstract

This study investigated the effect of* Eulophia macrobulbon* (EM) extract on sexual performance in aged-related erectile dysfunction (ED) rats. The ethanol EM extract at the doses of 15, 150, and 450 and sildenafil citrate at the dose of 5 mg/kg body weight (BW) were administered orally to the aged male rats once daily for 21 days. Mating parameters and intracavernosal pressure (ICP) were measured to evaluate their sexual and erection functions. Numbers of sperm and sperm motility as well as the diameter of seminiferous tubules were observed. The serum testosterone and 3',5'-cyclic guanosine monophosphate (cGMP) concentration in the rat penile tissue were analyzed. The results showed the significant increased sexual motivation, copulatory performance, and ICP of aged rats treated with sildenafil citrate and all doses of EM extract as compared to control aged rats. Moreover, their serum testosterone levels were slightly increased and significant increase in penile cGMP concentration was observed in these aged rats treated with sildenafil citrate and EM extract. The results suggest that treatment with EM could inhibit activity of PDE5 in penile tissue resulting in the increased cGMP level and bring to the improvement of erectile function and sexual performance.

## 1. Introduction

Erectile dysfunction (ED) refers to a man's consistent inability to achieve or maintain penile erection for satisfactory sexual activity [1]. The prevalence and the severity of ED increase with advancing age; different pathogenetic factors could contribute to age-related ED [2, 3]. Phosphodiesterase 5 (PDE5) protein expression and enzyme activity were significantly elevated in penile tissue of aged rats. Aged rats that received sildenafil citrate (a PDE5 inhibitor), an enzyme that promotes degradation of cGMP, showed a similar magnitude of intracavernosal pressure (ICP) as observed in the young group [4]. PDE5 inhibitors, sildenafil (Viagra®), vardenafil (Levirate®), tadalafil (Cialis®), and Avanafil (Stendra®) are commonly used for the treatment of ED [5, 6]. The most common side effect of PDE5 inhibitors is retinal defects through the inhibition of PDE6 [7, 8]. Therefore, the use of an herbal medicine having high selectivity could be an alternative to those synthetic PDE5 inhibitors.* Eulophia macrobulbon* (E.C. Parish & Rchb. f.) Hook. f. (EM) is a plant belonging to the Orchidaceae family. In Thailand, tuber of EM has been traditionally used for a long time to relieve pain and fatigue [9]. In previous study, it has been shown that the separation of EM tuber extracts yielded four phenanthrene compounds which were (1) 9,10-dihydro-4-(4′-hydroxybenzyl)-2,5-dimethoxyphenanthrene-1,7-diol, a new phenanthrene, (2) 1-(4′-hydroxybenzyl)-4,8-dimethoxyphenanthrene-2,7-diol, (3) 9,10-dihydro-2,5-dimethoxyphenanthrene-1,7-diol, and (4) 1,5,7-trimethoxyphenanthrene-2,6-diol). These phenanthrenes from EM were reported to serve as novel phosphodiesterase-5 inhibitors, and the most potent inhibitor was 1-(4′-hydroxybenzyl)-4,8-dimethoxyphenanthrene-2,7-diol which had IC_50_ value of 1.67±0.54 *μ*M [10]. Interestingly, 9,10-dihydro-4-(4′-hydroxybenzyl)-2,5-dimethoxyphenanthrene-1,7-diol showed mild activity on PDE5 with IC_50_ value of 62.26±32 *μ*M. However, EM has not been scientifically tested for its aphrodisiac activity on any animal models of ED. Therefore, it is of our interest to investigate whether standardized ethanolic EM extract could improve the erectile function in a rat model of aged-related ED.

## 2. Materials and Methods

### 2.1. Plant Material and Standardized* Eulophia macrobulbon* Extraction

EM was collected from Prachinburi Province, Thailand. The plant was identified by Asst. Prof. Dr. Anupan Kongbangkerd, Faculty of Sciences, Naresuan University. A voucher specimen (No. 002716) was deposited in the Biology Department, Faculty of Science, Naresuan University, Thailand. Ethanol extract of EM was prepared as described previously [10]. The extract was stored at -20°C until use. The ethanolic EM extract was standardized using 1-(4′-hydroxybenzyl)-4,8-dimethoxyphenanthrene-2,7-diol, as the standard markers and analyzed by the HPLC-UV/Vis system. The content of standard marker in the ethanolic extract of EM used in this study yields as 0.52%.

### 2.2. Animals and Housing

This study was conducted on adult (5-6-month-old) and naturally aged male rats (18-20-month-old). In addition, adult female (5-6-month-old) rats were used during sexual behavior testing. All animals were purchased from the National Laboratory Animal Center, Mahidol University, Salaya, Nakornpathom, Thailand. All experimental procedures conformed to the guidelines established by the National Research Council of Thailand and were approved by Naresuan University Animal Care and Use Committee (NUACUC) (approval no. 56 04 0064). Animals were housed in the standard animal housing conditions with maintained at a temperature of 22±1°C, a relative humidity of 55±10%, and a 12:12 h reversed light/dark cycle at Naresuan University Center for Animal Research (accreditation by the AAALAC International since 2014).

Male Wistar rats were divided into 6 groups, each group consisting of 10 animals as (1) young control, rats receiving the vehicle, propylene glycol (PG); (2) aged control, rats receiving PG; (3) aged + EM at 15 mg/kg BW; (4) aged + EM at 150 mg/kg BW; (5) aged + EM at 450 mg/kg BW; and (6) aged + sildenafil (5 mg/kg BW). The rats orally took either PG, EM, or sildenafil citrate daily for 21 consecutive days.

### 2.3. Procedures for Studying Sexual Behavior

At least 1 month before the behavioral testing, bilateral ovariectomy of female rats was performed with aseptic technique. The method of surgery is modified from that of Khajuria et al. in 2012 [11]. In brief, an adult female rat was deeply anesthetized using 2% isoflurane (2 volume percent supplemented by 1 L of oxygen per minute). After that, the fur covering the abdomen was completely shaved. Then a small (0.5–1 cm) midline incision was made on the abdomen in order to expose the peritoneal cavity. Then both ovaries were gently pulled, ligated, and removed from the abdomen. After that abdominal wall and the incision were sutured and povidone-iodine (Betadine®) was applied over and around the suture. The animals were allowed to fully recover from the surgery for 2 weeks to provide adequate time for endogenous hormone levels to diminish. Hormone replacement was used to induce estrous behavior in the female rats. Females were injected with 0.1 mg/kg BW estradiol benzoate (Sigma, St. Louis, USA) and 2 mg/kg BW progesterone (Sigma, St. Louis, USA) via subcutaneous route 48 h and 4 h before sexual behavior tests, respectively.

After dosing orally for 21 days, sexual behavior of all male rats were observed using a previously described method [12] in specially designed cages having glass on all sides and measuring 30 × 50 × 30 cu.cm. Each male rat was transferred to the cage and allowed to acclimatize in the box for 5 minutes before introducing an ovariectomized female rat in estrous phase. The sexual behaviors were constantly monitored and recorded by digital video recording for 30 minutes. All behavioral data such as mounting frequency (MF; the number of mounts without intromission in a session), mount latency (ML; the time interval between the introductions of the female and the first mount by the male), intromission frequency (IF; the number of intromissions in a session), intromission latency (IL; the time interval from the time of introduction of the female to the first intromission by the male), ejaculation frequency (EF; the number of ejaculations in a session), and ejaculatory latency (EL; the time interval between the first intromission and ejaculation) were scored according to published literature [13] by two observers blind to subject and treatment group.

### 2.4. Intracavernosal Pressure Measurement

Erectile function was assessed by measuring ICP next day after completion of sexual behavior test. Male rats were anesthetized with isoflurane at concentrations of 2-3%. Then the common carotid artery was cannulated with a polyethylene-50 (PE-50; 0.58 mm i.d. × 0.96 mm o.d.) tubing filled with heparin (250 IU/ml) to measure the mean arterial pressure (MAP). The crus of penis was inserted with a 23-needle connected to a PE-50 tubing filled with heparin to measure the ICP. Each PE-50 tubing was connected to a blood pressure transducer (model MLT0380/D, ADInstruments, Australia), transducer amplifier (Bridge Amp model ML142, ADInstruments), PowerLab 4/25T (ADInstruments), and a computer. A bipolar platinum electrode was placed around cavernous nerve. The electrical stimulation was delivered by stimulator at 2, 4, 6, or 8 volts with 20 Hz frequency for 1 min. to induce penile erection. All parameters were recorded using a LabChart program version 7.3.7 (ADInstruments). The pressure index was expressed as ICP/MAP. During the surgery for ICP measurement, the rat's condition was continuously monitored, the oxygen saturation by using a pulse oximetry and the body temperature by using a rectal probe which are connected to a PhysioSuite unit (Kent Scientific, USA).

### 2.5. Serum Testosterone Hormone Assay

After measurement of the erectile responses, blood samples were collected via cardiac puncture under deep anaesthesia and sent to BioLab Clinic (Phitsanulok, Thailand) for analysis of serum testosterone level.

### 2.6. Testicular Functions Assessment

Testicular function parameters assessed in this study were sperm count, sperm motility, and measurement of seminiferous tubule diameter. After completion of an ICP measurement, both sides of the cauda epididymis were excised, and semen was expelled and diluted with 1 ml of phosphate buffered saline (PBS) (pH 7.4, Gibco). 10 *μ*l of the sperm suspension was placed on haemocytometer (Counting Chamber, Makler®, USA). A total number and the percent motility of live sperm were counted. For assessment of the seminiferous tubular diameters, the left testis from each rat was collected, weighed, and preserved in 10% buffered formalin before conducting slide preparation with hematoxylin and eosin (H&E) staining. The diameters of seminiferous tubules per rat were randomly measured and calculated for the mean from 5 tubules. All of testicular function parameters were observed and assessed using a light microscope (Olympus BX43F, Japan; magnification: 20X, cellSens standard software).

### 2.7. Determination of cGMP Concentrations in Penile Tissue

After euthanizing the rat, the penis tissue was immediately removed, washed in cold PBS, weighed, rapidly frozen in liquid nitrogen, and stored at -80°C refrigerator until determination of cGMP concentrations. Penile tissue samples were assayed for cGMP levels by using an enzyme-linked immunoassay kit (Cayman Chemical Company, Ann Arbor, MI, USA).

### 2.8. Statistical Analysis

Effect of vehicle, EM, and sildenafil on mating behavior, ICP/MAP, and testicular functions was expressed as mean value ± standard error of the mean (SEM), whereas effect of vehicle, EM, and sildenafil on serum testosterone hormone level and penile cGMP concentration were expressed as mean value ± standard deviation (SD). Statistical analysis was performed using the statistical functions of the GraphPad Prism version 7.04. Statistical significance was determined by one-way analysis of variance (ANOVA) followed by Dunnett's test for multiple comparisons. A level of* P* value less than 0.05 was considered statistically significant.

## 3. Results

### 3.1. Effect of Ethanol Extract of* Eulophia macrobulbon* on Mating Behavior

There was an overall reduction in the sexual performance in the aged control rats as reflected by decrease in intromission frequency (IF) and increase in mount, intromission, and ejaculation latencies (ML, IL, and EL) as compared to the young control rats (Table 1). Aged rats receiving EM extract (15, 150, and 450 mg/kg BW) and sildenafil citrate (5 mg/kg BW) for 21 days showed a significant decrease in the ML, IL, and EL comparing to the aged control rats (*P *< 0.05). However, the IF of all EM-treated groups and sildenafil citrate-treated group was not significantly different from those of aged control group (*P *> 0.05).

### 3.2. Effect of Ethanol Extract of* Eulophia macrobulbon* on ICP

**Figure 1:**
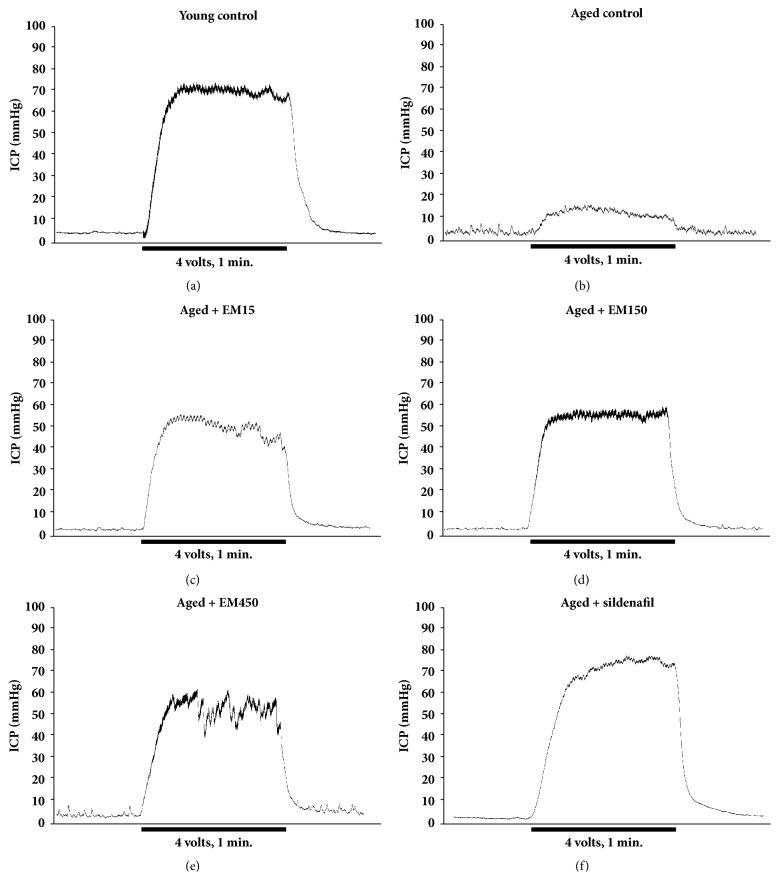
Representative recordings of ICP induced by cavernous nerve stimulation at 4 voltage, frequency of 20 Hertz for 1 min of each groups: (a) young control, (b) aged control, (v) aged + EM15 mg/kg BW, (d) aged + EM150 mg/kg BW, (e) aged + EM450 mg/kg BW, and (f) aged + sildenafil citrate 5 mg/kg BW.

**Figure 2:**
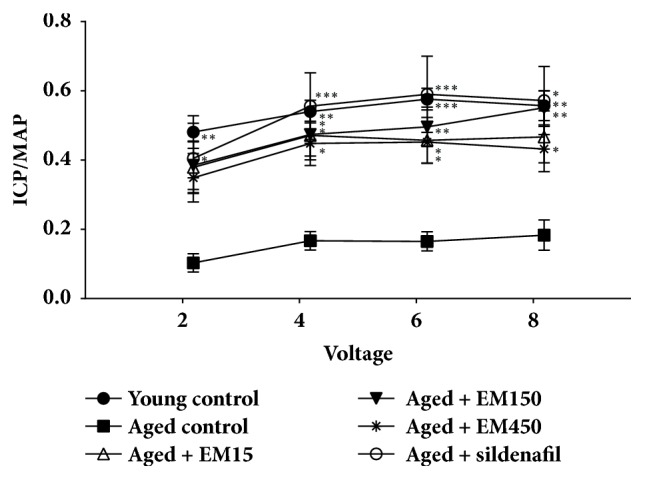
Effect of ethanol extract of EM on the intracavernosal pressure/mean arterial blood pressure ratio (ICP/MAP) in rats. Data are expressed as the mean ± SEM;* N*=10 of each group; ^*∗*^*P* < 0.05, ^*∗∗*^*P* < 0.01, and ^*∗∗∗*^*P* < 0.001 response significantly different compared with aged control group.

Treatment of aged rats with either EM extract or sildenafil showed increase in ICP which were statistically significant. The examples of ICP tracing in response to cavernous nerve stimulation are established in Figure 1. Treatment of the aged rats with the EM extract (15, 150, and 450 mg/kg BW) and sildenafil citrate (5 mg/kg BW) significantly induced increase in the pressure index compared with the aged control rats at all level of electrical stimulation (*P *< 0.05) (Figure 2).

### 3.3. Effect of Ethanol Extract of* Eulophia macrobulbon* on Serum Testosterone Level

**Figure 3:**
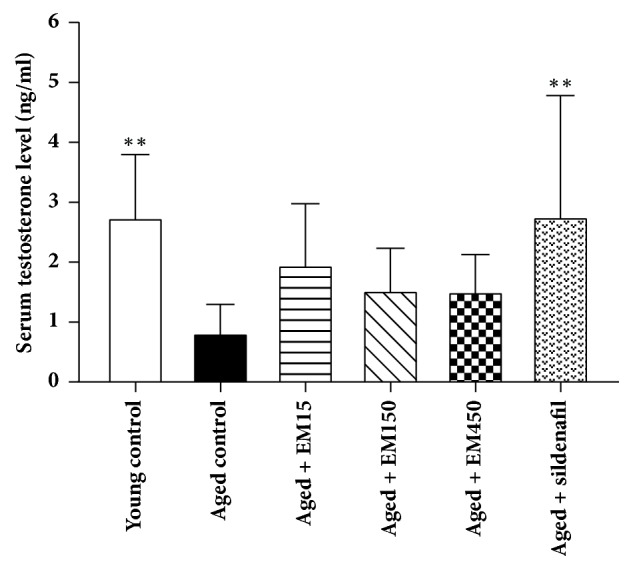
Effect of ethanol extract of EM on serum testosterone level of male rats. All values are expressed as mean ± SD; N = 10; ^*∗∗*^*P* < 0.01 significantly different compared with aged control group.

Testosterone level in young rats was significantly higher than that observed in aged control rats. Interestingly, in aged control rats treated with sildenafil citrate, the serum testosterone level (2.77 ± 2.02 ng/ml) was significantly increased (*P *< 0.01) when compared to aged control rats (0.83 ± 0.47 ng/ml). The increase in the serum testosterone levels was also observed in the aged rats treated with EM crude extract at the doses of 15, 150, and 450 mg/ kg BW (1.96 ± 1.02, 1.54 ± 0.70, and 1.52 ± 0.61 ng/ml, respectively) when compared with the aged control rats; however, these differences did not show statistical significance (*P* > 0.05) (Figure 3).

### 3.4. Effect of Ethanol Extract of* Eulophia macrobulbon* on Testicular Functions

**Figure 4:**
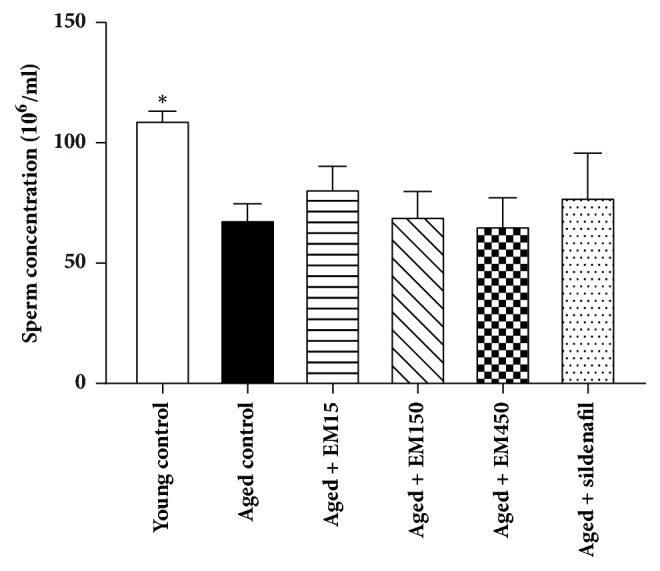
Effect of ethanol extract of EM on sperm concentrations in rats. Data are expressed as the mean ± SEM;* N*=10 rat per group, ^*∗*^*P* < 0.05 significantly different compared with aged control group.

**Figure 5:**
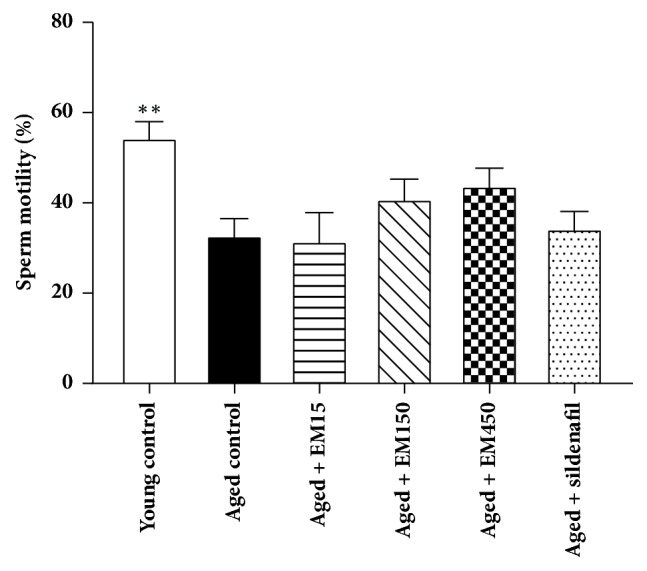
Effect of ethanol extract of EM on sperm motility in rats. Data are expressed as the mean ± SEM;* N*=10 rat per group, ^*∗∗*^*P* < 0.01 significantly different compared with aged control group.

**Figure 6:**
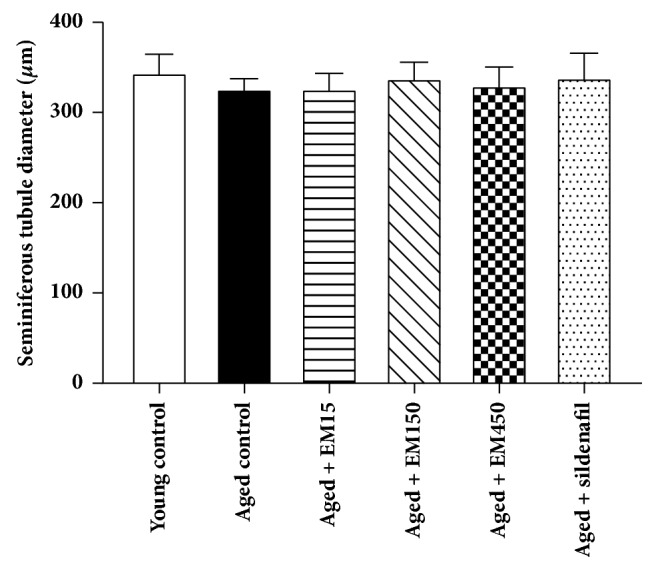
Effect of ethanol extract of EM on seminiferous tubule diameter in rats. Data are expressed as the mean ± SEM;* N*=10 rat per group.

Results of sperm concentration and motility level are shown in Figures 4 and 5, respectively. The young control group was significantly higher in the sperm concentration and percentage of motility when compared to the aged control rats. Administrations of EM extract at all doses or sildenafil citrate for 21 days did not alter the sperm concentration and motility (*P* > 0.05). A slight decline in the values mean of seminiferous tubules diameters was observed in the aged control group (328.20 ± 3.93 *μ*m) comparing with the young control group (346.25 ± 7.01 *μ*m) but this decline was not statistically significant (*P* > 0.05). Administrations of EM extract or sildenafil citrate for 21 days did not alter the diameters of seminiferous tubules (*P* > 0.05) in these aged rats when compared to the aged control rats (Figure 6).

### 3.5. Effect of Ethanol Extract of EM on cGMP Concentration in Penile Tissue

**Figure 7:**
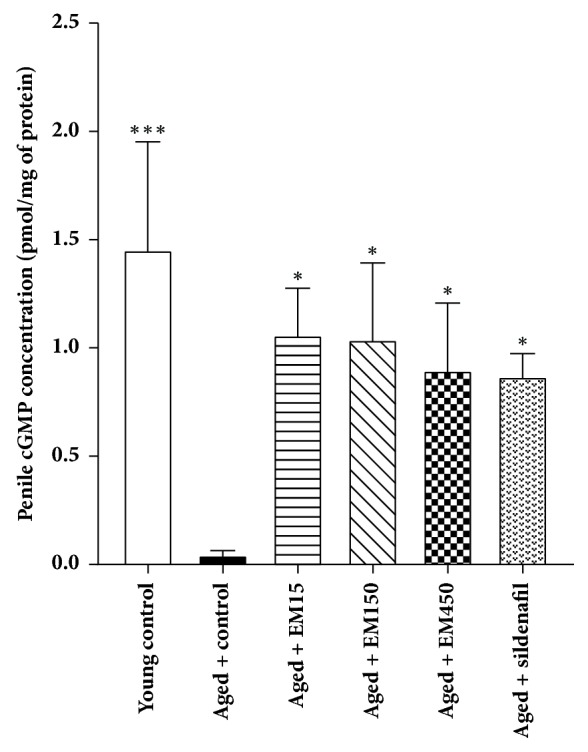
Effect of ethanol extract of EM on cGMP concentration in penile tissue of the experimental groups (mean ± SD);* N*=3 rats per group; ^*∗*^*P* < 0.05 and ^*∗∗∗*^*P* < 0.001 response significantly different compared with aged control group.

The effect of EM on cGMP accumulation in young and aged rat penile tissue is shown in Figure 7. The concentrations of cGMP were significantly reduced in penile tissue of aged rats (0.05 ± 0.02 pmol/mg of protein) compared with young rats (1.46 ± 0.50 pmol/mg of protein). When compared to aged control group, the cGMP concentrations in penile tissue of the aged rats treated with EM extract or sildenafil were significantly higher than that of the aged rats and no difference was observed when compared with the young control rats.

## 4. Discussion

This study examined the effect of EM extract on male sexual competence in aged male rats, with sildenafil citrate as positive reference drug. To the best of our knowledge, this is the first study to report that the ethanol extract from the tuber of EM could enhance the sexual motivation of male rats by decreasing the latencies of mounting and intromission significantly. Although the intromission frequency of aged rats treated with sildenafil and all doses of EM extract showed only slight increase that has no statistically significant difference, this may be an indication of improved efficiency of erection in the aged male rats. In addition, the slight increase in frequency and significant decrease in the latency of ejaculation in the aged rats administrated with the EM extract or sildenafil citrate indicated that the extract and drug have the potential to improve copulatory performance.

Male sexual behavior is highly dependent on testosterone, and the effect of testosterone on copulatory enhancement is mediated, in part, through permissive actions on DA release in the medial preoptic area (MPOA) [14]. Testosterone is secreted mainly by the Leydig cells. PDE5 is abundantly present in the rat Leydig and peritubular cells [15]. Administration with sildenafil, a PDE5-specific inhibitor, to the male mice for 4 weeks increased the testosterone production in Leydig cells, through cGMP accumulation due to PDE5 inhibition [16]. An in* vitro* study in Leydig cell also evidence that inhibition of PDE5 could lead to the increases of cGMP level and testosterone production [17]. It is well known that PDE5 plays a pivotal role on penile erection and both sildenafil citrate and EM extract have PDE5 inhibitory action. Our result agrees with previous study that, in the extract-treated groups, there was a slightly increased in serum testosterone levels, but it was a significant improvement in sexual performance [18]. It is possible that the improvement in sexual performance with the age could be due to the interaction of other mechanisms together with enhanced hormonal activity [19].

Penile erection is initiated by released nitric oxide (NO) from nonadrenergic noncholinergic (NANC) nerves and endothelial cells. NO then diffuses into cavernosal smooth muscle and activates guanylate cyclase, resulting in accumulation of cGMP and subsequent activation of PKG leading to the relaxation of cavernosal smooth muscle and penile erection [20]. This present study also evaluated the ICP in anesthetized rats as a direct index of penile rigidity and erectile function. The erectile response to cavernous nerve stimulation and penile cGMP level were significantly reduced in aged rats, which is consistent with the previous reports. There was a decrease in intracavernosal pressure response to electrical stimulation of the cavernosal nerve in aged rats (2–2.5 years old) due to reduction in cGMP concentration [21, 22] and smooth muscle: collagen ratio [23] in the penis. It is possible that the suppression effect of EM extract on PDE5 may enhance the cGMP-dependent vasodilation effect of NO leading to penile blood flow and erection. Additionally, sildenafil citrate-treated bilateral cavernous injury model of rats showed increase of smooth muscle level and decreased collagen deposition [24]. The present study in aged rats indicates that EM extract which contains phenanthrene compounds has aphrodisiac activity based on an inhibition action of PDE5 similar to sildenafil citrate.

The results of present study revealed a significant decline in sperm count and sperm motility and a slight decrease in seminiferous tubules diameters in the aged rats comparing with the young rats. It has been reported that regressed seminiferous tubules of male aged rats result from a low number of spermatogonia, spermatocytes, and spermatids that lead to decreased daily sperm production [25]. In addition, the serum testosterone concentration is necessary for sperm production and normal motility [26]. The results of the present study showed that sildenafil citrate had significant effect on serum T level but no significant effect on sperm count and motility in aged male rats. This was in accordance with previous study in which daily administration of sildenafil citrate at a higher dose (20 mg/kg) for 28 days to male aged rats (24-month-old) could insignificantly increase sperm count and motility [23].

## 5. Conclusion

Aging induced the decrease of cGMP concentration in penile tissue and serum testosterone level leading to the impairment of sexual behavior and erectile function. Treatment with* E. macrobulbon *extract for 21 days could reverse these effects by inhibition of PDE5 activity in smooth muscle of penile tissue resulting in the increased cGMP level and the improvement of sexual behavior and erectile function. Moreover, this extract may restore cGMP level in Leydig cells by inhibiting PDE5 activity resulting in the increased testosterone production. The present result provides, for the first time, information regarding the ability of EM extract to improve male sexual behavior and erectile function in aged rats. Therefore, EM extract may help to improve erectile dysfunction associated with aging.

## Figures and Tables

**Table 1:** Effects of 21-day administration of vehicle, EM (15, 150, and 450 mg/kg BW), or sildenafil citrate (5 mg/kg BW) on mating behavior in age-related ED rats.

**Group**	**Parameter**					
	**ML (min)**	**MF**	**IL (min)**	**IF**	**EL (min)**	**EF**
Young control	0.45 ± 0.13^*∗∗*^	11.70 ± 2.38	1.22 ± 0.24^*∗*^	48.50 ± 6.38^*∗∗∗*^	8.01 ± 2.53^*∗∗*^	1.20 ± 0.29
Aged control	1.55 ± 0.34	12.35 ± 4.31	5.39 ± 2.34	20.90 ± 3.80	27.93 ± 1.16	0.33 ± 0.21
Aged + EM15	0.55 ± 0.16^*∗∗*^	11.20 ± 3.59	0.77 ± 0.15^*∗*^	28.35 ± 3.11^##^	21.83 ± 3.09^*∗*^	0.85 ± 0.32
Aged + EM150	0.21 ± 0.05^*∗∗∗*^	7.20 ± 1.45	0.83 ± 0.37^*∗*^	27.40 ± 3.39^##^	19.83 ± 2.06^*∗∗*^	1.30 ± 0.29
Aged + EM450	0.55 ± 0.22^*∗*^	10.10 ± 3.03	1.13 ± 0.27^*∗*^	25.50 ± 1.70^##^	20.99 ± 2.34^*∗*^	1.40 ± 0.33
Aged + sildenafil	0.45 ± 0.12^*∗∗*^	11.55 ± 2.29	1.15 ± 0.25^*∗*^	29.20 ± 2.68^##^	20.13 ± 2.52^*∗∗*^	1.20 ± 0.29

ML: mount latency; MF: mount frequency; IL: intromission latency; IF: intromission frequency; EL: ejaculation latency; EF: ejaculation frequency. Latencies are expressed in minutes (min), and frequencies are expressed as number of events. Data are expressed as the mean ± SEM; N=10; ^*∗*^*P* < 0.05, ^*∗∗*^*P *< 0.01, and ^*∗∗∗*^*P* < 0.001 response significantly different compared with aged control group; ^##^*P* < 0.01 response significantly different compared with young control group.

## Data Availability

The data that support the findings of this study are available from the corresponding author upon reasonable request.
